# Toll-Like Receptor 9 Inactivation Alleviated Atherosclerotic Progression and Inhibited Macrophage Polarized to M1 Phenotype in ApoE^−/−^ Mice

**DOI:** 10.1155/2015/909572

**Published:** 2015-07-15

**Authors:** Chunmei Ma, Qiufang Ouyang, Ziyang Huang, Xiaoqing Chen, Ye Lin, Weiping Hu, Ling Lin

**Affiliations:** ^1^Cardiovascular Department, Second Affiliated Hospital and Second Clinical Medical College, Fujian Medical University, Quanzhou, Fujian 362000, China; ^2^Key Laboratory of Cardiovascular Remodeling and Function Research, Chinese Ministry of Education and Chinese Ministry of Health, Shandong University Qilu Hospital, Jinan, Shandong 250012, China; ^3^Rheumatism Department, Second Affiliated Hospital and Second Clinical Medical College, Fujian Medical University, Quanzhou, Fujian 362000, China

## Abstract

*Objective*. Toll-like receptor 9 (TLR9) is involved in many inflammatory diseases, but its role in atherosclerosis remains controversial. This study aimed to investigate the role of TLR9 in atherosclerosis development and macrophage polarization. *Methods*. ApoE^−/−^ mice were treated with vehicle or IRS869 for 12 weeks. Plaque vulnerability was assessed with immunohistochemical analysis, picro-sirius red, and oil red O staining. The expressions of M1- and M2-associated markers in plaques were detected by RT-PCR and immunofluorescence. The aorta TLR9 and its downstream molecules including myeloid differentiation protein 88 (MyD88), phosphorylated nuclear factor-kappa B (p-NF-*κ*B), and interferon regulatory factor 7 (IRF7) were determined by western blot analysis. The frequency of M1 and M2 subtype in RAW264.7 cells treated with IRS869 and/or ODN1826 was evaluated with flow cytometry. *Results*. In ApoE^−/−^ mice, functional inactivation of TLR9 pathway resulted in attenuated atherosclerosis development, as manifested by reduced plaque burden and by decreased plaque vulnerability. Mechanistically, TLR9 inhibition prevented the activation of MyD88/NF-*κ*B pathway and shifted the balance of M1/M2 toward M2 macrophages that were involved. *Conclusions*. Our data indicated that TLR9 inactivation ameliorated atherosclerosis via skewing macrophage plasticity to M2 phenotype in ApoE-deficient mice. These findings may provide a promising therapeutic strategy for atherosclerosis.

## 1. Introduction

Atherosclerosis is a chronic inflammatory disease of blood vessels, of which macrophages play essential roles in the development of atherosclerosis. Depending on the microenvironment, macrophages can acquire distinct functional phenotypes, referred to as classically activated, proinflammatory macrophages (M1) and alternatively activated, anti-inflammatory macrophages (M2). Macrophages are polarized to the M1 phenotype by exposure to T helper cell type I (Th1) cytokines or in the presence of bacterial products such as lipopolysaccharide (LPS). And M2 macrophages are polarized by stimulation with Th2 cytokines such as interleukin- (IL-) 4 and IL-13. The levels of IL-1*β*, inducible nitric oxide synthase (iNOS), and MHC class II transactivator (Ciita) are upregulated in M1 macrophages while the expressions of found in inflammatory zone 1 (FIZZ1), arginase-1, and Ym1 are upregulated in M2 cells. Macrophage polarization is plastic, suggesting that the M1 to M2 switch enables the dual role of macrophages in orchestrating the development of atherosclerosis [1].

Macrophages sense the endogenous danger signals through pattern-recognition receptors, and toll-like receptors (TLRs) are the most common pattern-recognition receptors families [2]. TLR9 is an endosomal protein that recognizes both bacterial DNA and self-DNA. ODN1826 is a type B CpG oligodeoxynucleotide (ODN) which can activate mouse TLR9 [3], while IRS869, a special antagonist of TLR9, can inhibit the expression of TLR9 and related cytokine. TLR9 detects unmethylated CpG DNAs or CpG oligodeoxynucleotide (ODN). Upon the ligation with its cognate ligands, TLR9 recruits the MyD88 adapter protein; then the nuclear factor *κ*B- (NF-*κ*B-) dependent proinflammatory cytokines pathway and the interferon regulatory factor 7- (IRF7-) dependent type I interferons (IFNs) pathway are initiated [4]. Most studies hitherto have focused on the implications of TLR4 or TLR2 on atherosclerosis, while the data on the roles of TLR9 in atherosclerosis is scarce. TLRs have been considered to be the major culprits in the development of atherosclerosis, contributing to both its progression and clinical complications. However, the direct evidence for the effect of blocking TLR9 on accelerated atherosclerosis is lacking.

Therefore, in the present study, we aimed to determine whether TLR9 signaling can directly regulate the macrophage polarization and promote the progression of atherosclerosis. We demonstrate that IRS869 can skew M1/M2 balance to M2 phenotype and alleviate the vulnerability of the atherosclerotic plaques in ApoE^−/−^ mice.

## 2. Materials and Methods

### 2.1. Animals and Experimental Design

All of the procedures and protocols were approved by the Animal Care Committee of Fujian Medical University and followed the guidelines of Animal Management Rules of the Chinese Ministry of Health. Twenty 12-week-old female ApoE^−/−^ on C57BL/6 background mice were obtained from Peking University Animal Center (Beijing, China). The mice were randomised into two groups: control saline mice and IRS869 treated mice. Mice in these groups received intraperitoneal injections of vehicle or IRS869 (5 mg/kg, 5′-TGCTTGCAAGCTTGCAA GCA-3′; Shenggong Company) twice a week for 12 weeks according to the reports [5, 6]. The wild-type mice on C57BL/6 background treated with saline served as normal controls (*n* = 10). Each group was fed on a western-type diet (containing 0.25% cholesterol and 15% cocoa butter). They were maintained under standardised lighting conditions (12 h light-dark cycle) and temperature (21 ± 1°C), and mineral water was administered* ad libitum*. At the end of 12 weeks, the mice were deprived of food for 8 hours and killed with an overdose of pentobarbital (50 mg/kg). Specimens of serum, brachiocephalic artery, aortic root, and aorta were collected.

### 2.2. Immunohistochemical Analysis, Picrosirius Red, and Oil Red O Staining for Plaque Vulnerability

The brachiocephalic artery was serially cross-sectioned into 5 *μ*m thick pieces at 50 *μ*m intervals. For plaque vulnerability analysis, immunohistochemistry was performed to detect macrophage marker CD68 (Abcam, 1 : 200) and smooth muscle *α*-actin (Santa Cruz, 1 : 100). The content of type I collagen was assessed by picrosirius red staining, and the deposition of lipids was determined with oil red O staining. The plaque vulnerability index was calculated as positive staining area of (macrophages + lipid) divided by positive staining area of (smooth muscle *α*-actin + collagen) [7].

### 2.3. Western Blot Analysis for TLR9, MyD88, p-p65-NF-*κ*B, and IRF7 in Aorta

Western blot analysis was performed to determine the TLR9, MyD88, phosphorylated nuclear factor-kappa B (p-NF-*κ*B), and IRF7 in the aorta. Primary antibodies from Abcam were used at the indicated dilutions as follows: anti-TLR9 (1 : 500), MyD88 (1 : 1000), total NF-*κ*B antibody (1 : 1000), p-NF-*κ*B (1 : 500), and IRF7 (1 : 1000). After washing, bound antibody was detected using anti-rabbit antibody linked to horseradish peroxidase and bound complexes were detected using enhanced chemiluminescence. The intensity of the bands was quantified by densitometry. Blots were representative of at least three experiments showing the same results.

### 2.4. Real Time PCR (RT-PCR) for Macrophages M1 and M2 Subsets in Plaques

Total RNA isolated from aorta was treated with DNase I at 37°C for 30 min before reverse transcription was performed using a high capacity cDNA archive kit (TaKaRa, Japan). The PCR was performed with the MasterMix System (Roche, Switzerland). The sequences of primers (5′ to 3′) were as follows: (1) for IL-1*β*: CAACCAACAAGTGATATTCTCCATG (forward) and GATCCACACTCTCCA GCTGCA (reverse); (2) for iNOS: ACCCACATCTGGCAGAATGAG (forward) and AGCCATGACCTTTCGCATTAG (reverse); (3) for Ciita: CTCAGCCTTAGG GACTGG (forward) and GACCTGGATCGTCTCGTGCAG (reverse); (4) for IL-10: GCTCTTACTGACTGGCATGAG (forward) and CGCAGCTCTAGGAGCATGTG (reverse); (5) for Ym1: CAAGTTGAAGGCTCAGTGGCTC (forward) and CAAATCATTGTGTAAAGCTCCTCTC (reverse); (6) for Fizz1: CCCTCCA CTGTAA CGAAGACTC (forward) and CACACCCAGTAGCAGTCATCC (reverse); (7) for GADPH: AGGTCGGTGTGAACGGATTTG (forward) and TGTAGACCATG TAGTTGA GGTCA (reverse). The relative quantification of the target gene mRNA used the comparative ΔΔCT-method, normalised to an endogenous reference (GADPH) and a relevant normal control equal to 2^−ΔΔCT^. The expression levels of target genes were determined in triplicate from the standard curve.

### 2.5. Immunofluorescent Staining for iNOS and CD206 in Plaques

For the localisation of the iNOS^+^ and CD206^+^ cell in plaques, immunofluorescence staining for iNOS^+^ (1 : 150; Abcam) and CD206^+^ (1 : 100; Abcam) was performed in the brachiocephalic artery plaques. And the secondary antibodies were fluorescein isothiocyanate (FITC) or Alexa Fluor 546, respectively. The number of iNOS^+^ and CD206^+^ cells was expressed as a fraction of the total cell nuclei.

### 2.6. Cell Cultures of RAW264.7 Macrophages 

The mouse macrophage cell line RAW264.7 (ATCC, Rockville, MD) (2.5 × 10^5^/mL) was cultured in Dulbecco's modified Eagle's medium (DMEM) supplemented with 10% (vol/vol) heat-inactivated fetal bovine serum (GIBCO, Chagrin Falls, IL), streptomycin (100 *μ*g/mL), and penicillin (100 U/mL) at 37°C in a humidified atmosphere with 6% CO_2_. The number and viability of macrophage cells were assessed by trypan blue dye exclusion using a hematocytometer. Prior to treatment, cells (2.5 × 10^5^/mL) were cultured for 24 h to achieve 80% confluence. Then RAW264.7 cells were treated for 24 h with 5 *μ*M ODN1826 (InvivoGen San Diego, CA, USA,) and/or IRS869 (5 *μ*M).

### 2.7. Flow Cytometric Detection of iNOS^+^ and CD206^+^ Cells

Stimulated RAW264.7 macrophages were removed from wells and incubated in mouse BD Fc Block (BD Biosciences, San Jose, CA) for 30 min on ice. Samples were divided and aliquots stained using anti-CD206-phycoerythrin (PE) and anti-iNOS-fluorescein isothiocyanate (FITC) (all purchased from BD Biosciences, San Jose, CA). Percentage of M1 (iNOS^+^) and M2 (CD206^+^) cells was determined by flow cytometry on a FACSCalibur (BD Biosciences, Heidelberg, Germany) and analyzed by FlowJo software (Tree Star Inc., Ashland, OR, USA).

### 2.8. Serum Lipid Analysis

The fasting serum samples were collected at the end of experiment. The total cholesterol (TC), triglycerides (TG), high-density lipoprotein cholesterol (HDL-C), and non-HDL-C were measured as previously described [8].

### 2.9. Statistical Analysis

All values were expressed as the mean ± SE unless otherwise indicated. Student's *t*-test (two-sample test) or one-way analysis of variance was performed to assess the difference between groups by using SPSS 13.0. The Mann-Whitney *U* test was used if the data was not normally distributed. All reported *P* values were two-tailed, and a *P* value of 0.05 was regarded as significant.

## 3. Results

### 3.1. Inactivation of TLR9 Attenuated Atherosclerotic Plaque Area in ApoE^−/−^ Mice

**Figure 1:**
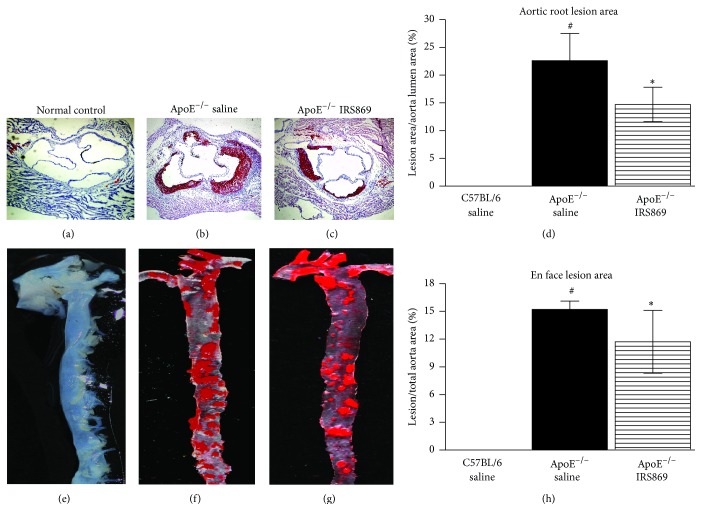
Inactivation of TLR9 attenuated atherosclerotic plaque burden. Representative oil red O stained photomicrographs from aortic root (a–c) and from en face preparations of the aortic tree (e–g). Histogram represented mean ± SEM from aortic root (d) and aortic tree (f) in 7 mice. ^#^ *P* < 0.001 versus normal control mice, ^*∗*^ *P* < 0.05 versus ApoE^−/−^ saline mice.

To determine the effect of IRS869 on atherosclerotic plaque development, we quantified plaque area in the aortic root (Figures 1(a)–1(d)) and aorta tree (Figures 1(e)–1(h)) by oil red O staining. No plaque was detected in wild-type normal control mice. IRS869 treatedmice revealed a significant reduction in lesion size in comparison with control mice. The plaque burden of IRS869 ApoE^−/−^ was decreased by 34.9% in aortic root and by 23.0% in the whole aorta tree as compared to ApoE^−/−^ controls.

### 3.2. Functional Inactivation of TLR9 Alleviated Atherosclerotic Vulnerability in ApoE^−/−^ Mice

**Figure 2:**
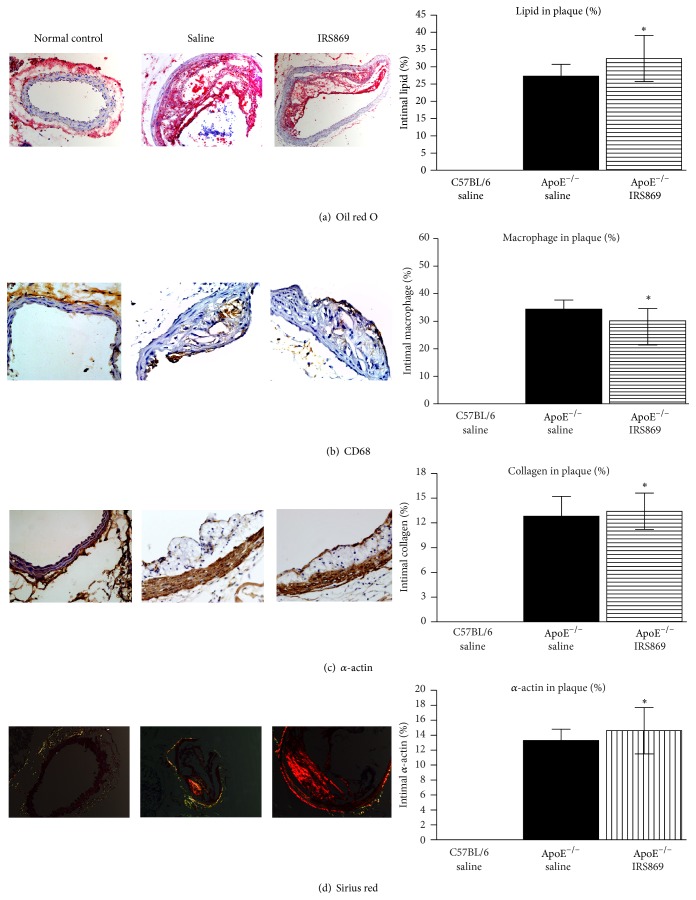
Inactivation of TLR9 alleviated atherosclerotic vulnerability. Oil red O staining of lipids (a), immunostaining for CD68 positive macrophages (b) and *α*-actin (c), and sirius red staining for collagen (d) in brachiocephalic artery plaques. Bars represent mean ± SEM from 7 mice. ^*∗*^ *P* < 0.05 compared with ApoE^−/−^ saline mice. Original magnification ×200.

Since the brachiocephalic artery is the most common site of plaque rupture in ApoE^−/−^ mice [9], plaque vulnerability in the brachiocephalic artery was determined. Histological analysis revealed IRS869 treated mice displayed profound changes in plaque composition (Figure 2). The content of collagen and smooth muscle cell was obviously elevated as compared to ApoE^−/−^ controls. Additionally, macrophage infiltration in plaques, as assessed by CD68^+^ macrophages, was significantly alleviated by IRS869 treatment. Accordingly, the plaque vulnerability index was decreased from 3.7 to 2.6.

### 3.3. Decreased TLR9, MyD88, p-p65-NF-*κ*B, and IRF7 Expression in the Aortic Plaques after IRS869 Treatment

**Figure 3:**
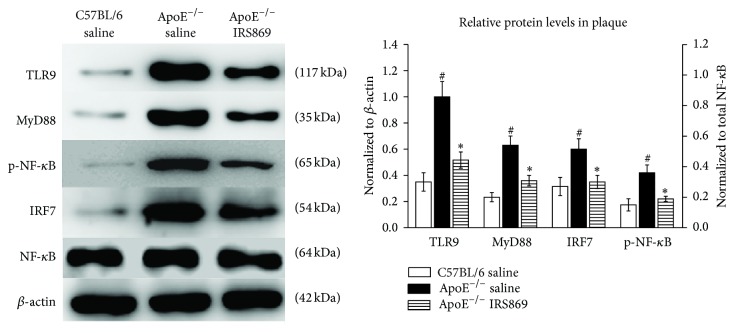
Effects of IRS869 on the expression of TLR9 and its major downstream molecules in plaques. The expressions of TLR9, MyD88, p-NF-*κ*B, and IRF7 were determined by western blotting. *β*-actin (for TLR9, MyD88, and IRF7) or NF-*κ*B (for p-NF-*κ*B) served as the loading control. The bars represent the mean ± SEM of three separate experiments. ^#^<0.01 compared with normal control littermates, ^*∗*^ *P* < 0.05 compared with ApoE^−/−^ saline controls. TLR9: toll-like receptor 9; MyD88: myeloid differentiation protein 88; NF-*κ*B: nuclear factor-kappa B; p-NF-*κ*B: phosphorylated NF-*κ*B; IRF7: interferon regulatory factor 7.

The molecules of NF-*κ*B, MyD88, and IRF7 were the critical downstream signaling components in TLR9 pathway. To determine whether those molecules were involved during TLR9 inactivation, the protein levels of TLR9 and the downstream molecules (MyD88, NF-*κ*B, and IRF7) were determined by western blotting. The expressions of those molecules were significantly upregulated in ApoE^−/−^ saline mice compared to normal controls. And the levels of TLR9, MyD88, p-NF-*κ*B, and IRF7 were markedly lower in ApoE^−/−^ IRS869 mice than those in the ApoE^−/−^ saline littermates (Figure 3).

### 3.4. TLR9 Inactivation Inhibited M1 Macrophage Infiltration in Atherosclerotic Plaques

**Figure 4:**
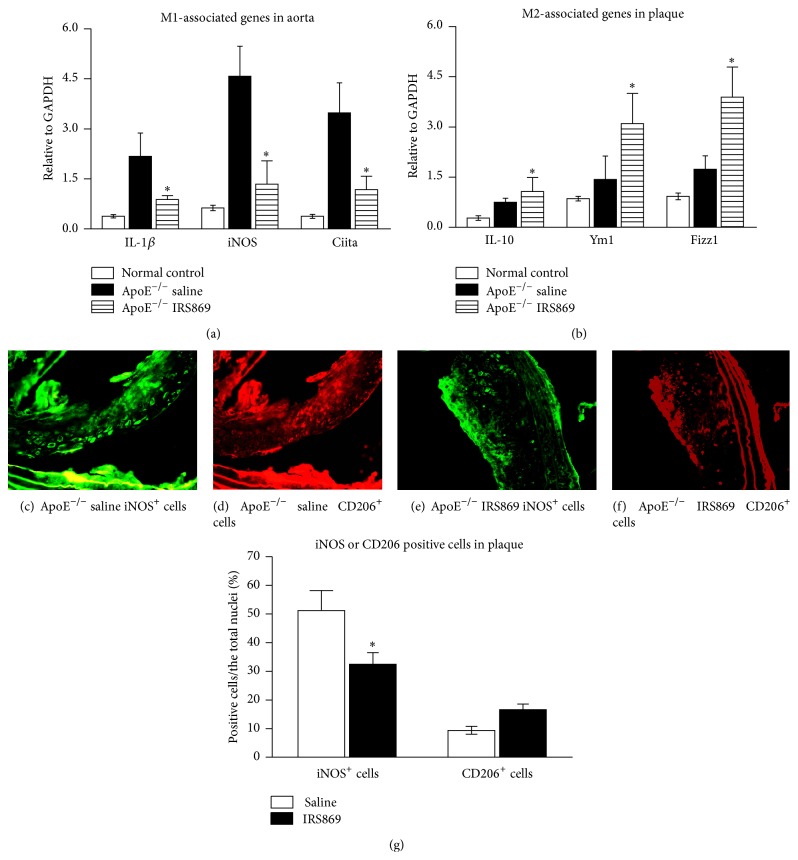
TLR9 inactivation inhibited M1 macrophage infiltration. RT-PCR for the mRNA expressions of M1- (a) and M2- (b) associated genotype in ApoE^−/−^ mice treated with IRS869. The relative gene expression was normalized to GAPDH. Representative fluorescent photomicrographs of iNOS (d and f, green) and CD206 (e and g, red) stained sections in brachiocephalic artery plaques. Results showed mean ± SEM of three separate experiments. ^*∗*^ *P* < 0.05 compared with ApoE^−/−^ saline mice. Original magnification ×200. IL: interleukin; Ciita: MHC class II transactivator; iNOS: inducible nitric oxide synthase; Fizz1: found in inflammatory zone 1.

To investigate the mechanism by which TLR9 modulates atherosclerosis, we analyzed M1/M2 macrophage polarization in plaques. Aorta total RNA was extracted; then the expression of M1-associated (IL-1*β*, iNOS, and Ciita) and M2-related genes (IL-10, Ym1, and Fizz1) was determined by RT-PCR (Figures 4(a) and 4(b)). As compared to control mice, IRS869 treatment caused a powerful inhibition of M1 signature mRNA expression such as IL-1*β*, iNOS, and Ciita. Meanwhile, the expressions of M2 markers Fizz1 and Ym1 were upregulated, excepting IL-10 (which was marginally increased).

To further determine whether the mRNA expression patterns changed uniformly at the protein level, the distribution of iNOS^+^ (M1 marker, green) and CD206^+^ (M2 marker, red) macrophages was localized with immunofluorescent staining. IRS869 treated mice exhibited a strikingly lower iNOS^+^ while exhibiting slightly higher CD206^+^ macrophage infiltration in plaques compared with control mice (Figures 4(c)–4(g)). Those data indicated that TLR9 inactivation inhibited M1 macrophage infiltration in atherosclerotic plaques.

### 3.5. TLR9 Inactivation Was Associated with a Reduction in M1 and Increased M2 Cells in RAW264.7 Macrophages

**Figure 5:**
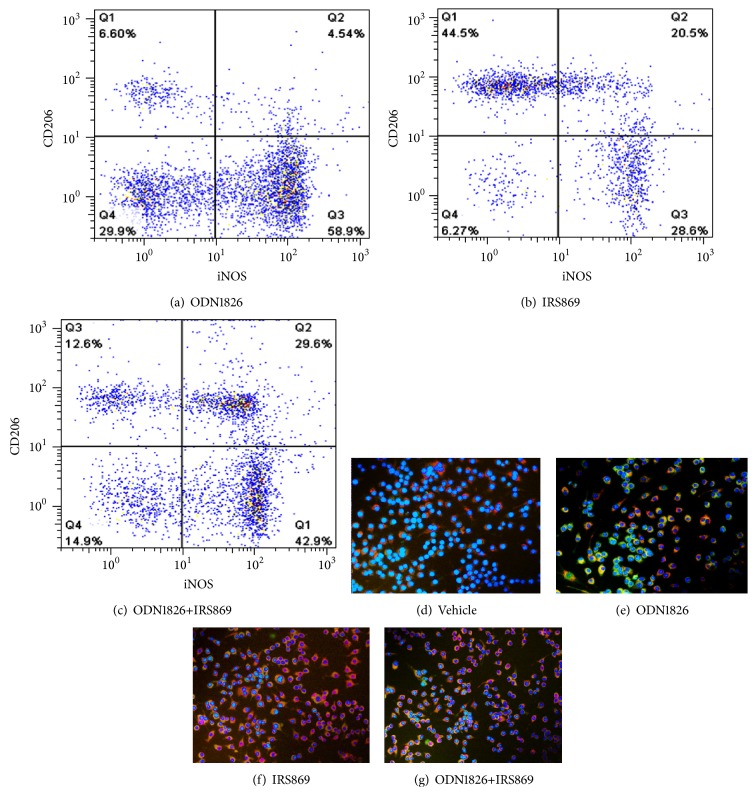
Effects of ODN1826 and IRS869, alone or in combination, on the percentages of M1 and M2 subtype in RAW264.7 macrophages. Representative flow cytometry analyses of M1 and M2 cells and double immunofluorescence labeling of iNOS and CD206 macrophages in RAW264.7 cells. RAW264.7 cells were treated for 24 h with 5 *μ*M ODN1826 (a, e), 5 *μ*M IRS869 (b, f), ODN1826+IRS869 (c, g), or vehicle (d). The cells were stained with PE-conjugated anti-CD206 (red) and FITC-conjugated anti-iNOS (green) monoclonal antibodies. Nuclei were stained with DAPI (blue).

To investigate whether TLR9 activation had a direct effect on macrophages skewness* in vitro*, RAW264.7 macrophages were treated with TLR9 ligand C-phosphate-G (CpG) oligodeoxynucleotide ODN1826 (TLR9 agonist) and/or IRS869 (a special antagonist of TLR9). The percentage of M1 or M2 macrophages in the total cell population was determined by flow cytometry. In RAW264.7 macrophages, ODN1826 induced a remarkably stronger M1 response, as manifested by the significantly increased iNOS^+^ macrophages, while IRS869 notably increased the proportion of CD206^+^ cells. Coadministration of ODN1826 and IRS869 caused a significant reduction in the percentage of M1, whereas the production of M2 was not altered as compared with those cells treated with ODN1826 (Figures 5(a)–5(c)).

To further validate the results, those cells were examined by double labeled immunofluorescence for iNOS and CD206. Consistent with the flow cytometric analysis outcomes (Figures 5(d)–5(g)), the frequency of iNOS^+^ cells (green) was significantly higher than CD206^+^ cell (red) in RAW264.7 cells triggered with ODN1826, whereas, in cells treated with IRS869, the vast majority of cells were CD206 positive. Consequently, these results suggested that TLR9 inactivation shifts the balance of M1/M2 to M2 phenotype skewness in RAW264.7 cells.

### 3.6. Serum Lipid Levels

The serum lipids were detected to evaluate the effects of TLR9 inhibition on the lipid profiles of the experimental mice. Compared with the normal control mice, the serum cholesterol, triglycerides, and non-HDL-C levels were markedly elevated in the ApoE^−/−^ genotype mice. And the serum levels of total cholesterol and triglycerides in the IRS869 treated group (19.61 ± 3.54 mmol/L and 2.93 ± 4.46 mmol/L, resp.) did not differ significantly from those in the nontreatment ApoE^−/−^ mice (20.01 ± 3.26 mmol/L and 2.84 ± 0.35 mmol/L, resp.) (Table 1).

## 4. Discussion

This study investigated, for the first time, the direct effects of TLR9 inactivation on atherosclerotic plaque progression in ApoE^−/−^ mice and macrophage polarization* in vivo* and* in vitro*. Our data demonstrated that TLR9 inactivation caused not only a quantitative decrease in plaque burden, but also a qualitative change in plaque composition to more stable lesions. Mechanistically, TLR9 inhibition prevented the activation of MyD88/NF-*κ*B pathway and shifted the balance of M1/M2 to M2 skewness involved. Our findings were partially substantiated by the reports that the antagonists of TLR7 and TLR9 reduced postinterventional remodeling by preventing neointima formation and accelerated atherosclerosis [10] and chloroquine (a TLR9 inhibitor) induced atheroprotection [11]. Our data showed that TLR9 inhibition in ApoE^−/−^ mice displayed a modest decrease (34.9% in aortic root and 23.0% in the whole aorta tree) in the extent of atherosclerosis. And there was evidence indicating that MyD88^−/−^ApoE^−/−^ mice displayed a 57% reduction in the extent of atherosclerosis [12]. This finding suggests that TLR9 is one contributor to, but not the single cause of, mediating downstream proatherogenic effects via MyD88 signaling.

Vulnerable plaques are the primary cause of cardiovascular and cerebrovascular diseases. Vulnerable plaques are characterized by accumulation of lipid laden foam cells and macrophages and by decreased collagen and smooth muscle cell. Our results indicated the lipid content in control mice was lower than that in IRS869 treated mice; it may be due to large areas of necrosis observed in ApoE^−/−^ vehicle mice.

Atherosclerosis is a chronic inflammatory disease. And macrophages are the most important inflammatory cell in the development of atherosclerosis. Macrophage has plasticity and can switch between the M1 and M2 phenotypes. Classically (M1) and alternatively (M2) activated macrophages represent the two extremes of a continuum of macrophages activation states. Compelling evidence demonstrated that macrophage polarization played a great role in the stability of atherosclerosis plaques [13]. M1 macrophages were associated with vulnerable plaques, while M2 macrophages enhanced the stability of atherosclerotic plaques [14]. M2 macrophages were infiltrated in early lesions of ApoE^−/−^ mice, while M1 macrophages were predominant in advanced plaques of aged animals [15]. Our data indicated that TLR9 inactivation resulted in greater polarization towards an M2 phenotype as evidenced by increased ratio of M2-to-M1 macrophages. Notably, our finding is that ApoE^−/−^ mice treated with IRS869 exhibited slightly increased M2 macrophages in plaques, which seemingly contradicted our result* in vitro* that RAW264.7 macrophages treated with IRS869 induced substantially increased M2 cells. The possible explanation for this discrepancy might be the ability to restore the self-equilibrium to avoid excessive proinflammatory reaction* in vivo*.

The role of TLR9 in atherosclerosis is quite controversial. It is reported that CpG ODN activates the TLR9-MyD88-ERK1/2 pathway, thus inducing foam cell formation [16]. Additionally, ODN1826, the agonist ligand of TLR9, can significantly enhance perilipin 3 expression and macrophage accumulation of lipids, especially triglycerides in RAW264.7 cells [17]. Alternatively, compelling evidence suggested that MyD88-dependent TLR signaling plays an important role in the development of atherosclerotic plaques and the activation of TLR9 facilitated the formation of foam cells in an NF-*κ*B- and IRF7-dependent manner [18, 19]. Consistent with those findings, our results indicated that inactivation of TLR9 downregulated MyD88, p-p65-NF-*κ*B, and IRF7 and alleviated atherosclerosis progression, given that antibodies to RNA- or DNA-containing autoantigens are characteristic of systemic lupus erythematosus (SLE). Our work could help unravel the mechanism that accelerated atherosclerosis in patients with SLE. However, our data is in contrast to the report that genetic deletion of TLR9 exacerbates atherosclerosis and TLR9 exerts atheroprotection in ApoE^−/−^ mice [6]. The possible explanation for this discrepancy might be the difference in gender and treatment time. This argument was supported by the report that there were significant differences in cytokine, plaque sizes, and content of smooth muscle cells between male and female ApoE^−/−^ mice [20].

In conclusion, our data demonstrated the novel observations that TLR9 inactivation skewed the balance of M1/M2 macrophages toward the M2 phenotype and reduced plaque vulnerability. Our study may be valuable for deciphering the cross talk between the autoimmune response and atherosclerosis and provide a promising therapeutic strategy for the atherosclerosis, given that atherosclerosis is a multifactorial disease in which diverse mechanisms are involved. Thus the roles of other TLRs and other kinds of macrophages phenotypes in atherosclerosis thus warrant further examination.

## Figures and Tables

**Table 1:** Lipid profile of the ApoE^−/−^ and wild-type mice fed on the western-type diet.

	TC (mmol/L)	TG (mmol/L)	Non-HDL (mmol/L)	HDL-C (mmol/L)
Normal control	3.25 ± 0.48	1.03 ± 0.24	1.71 ± 0.37	1.47 ± 0.54
ApoE^−/−^ saline	20.01 ± 3.26^#^	2.84 ± 0.35^#^	18.83 ± 3.1^#^	0.32 ± 0.23^#^
ApoE^−/−^ IRS869	19.61 ± 3.54^#^	2.93 ± 4.46^#^	17.91 ± 4.34^#^	0.35 ± 0.71

The results were expressed as the mean ± SE. ^#^<0.01 compared with normal control littermates; TC: total cholesterol; TG: triglycerides; HDL-C: high-density lipoprotein cholesterol.
